# Nursing Interventions Supporting Rehabilitation and Functional Improvement in Older Adults with Coronary Heart Disease: A Scoping Review

**DOI:** 10.3390/healthcare14213794

**Published:** 2026-11-06

**Authors:** Alvaro Ortega-Maldonado, Anabel Chica-Perez, Dulcenombre de Maria García-Lopez, Miguel Gerez-De-Paco, Cayetano Fernandez-Sola, Jose Manuel Hernandez-Padilla

**Affiliations:** 1 Santa Ana Hospital, 18600 Motril, Spain; aom553@inlumine.ual.es; 2 Department of Nursing, Physiotherapy and Medicine, University of Almeria, 04120 Almeria, Spain; cfernan@ual.es (C.F.-S.); j.hernandez-padilla@ual.es (J.M.H.-P.); 3 Torrecárdenas University Hospital, 04009 Almeria, Spain; dgl896@inlumine.ual.es; 4 La Inmaculada Hospital, 04600 Huercal-Overa, Spain; mgd461@ual.es; 5 Facultad de Ciencias de la Salud, Universidad Autónoma de Chile, Santiago 7500000, Chile

**Keywords:** chronic heart disease, older adults, nursing, cardiac rehabilitation, healthcare services, continuity of care, transitional care

## Abstract

**Background/Objectives**: Coronary heart disease (CHD) is a major cause of morbidity, disability, and loss of autonomy among older adults. Nursing interventions may support rehabilitation and continuity of care, although their characteristics and delivery remain heterogeneous. This scoping review aimed to map nursing interventions implemented and evaluated to support rehabilitation and functional improvement in older adults with CHD, focusing on how these interventions were organised and delivered, the roles assumed by nurses, and the outcomes assessed. **Methods**: A scoping review was conducted following Joanna Briggs Institute’s guidance and reported according to PRISMA-ScR. PubMed, CINAHL, Scopus, Web of Science, and Cochrane were searched between November 2025 and February 2026. Eligibility criteria followed the Population–Concept–Context framework. Quantitative and mixed-method intervention studies involving adults with CHD with a mean age ≥60 years were included. Study selection was performed independently by two reviewers. **Results**: Twelve studies were included. Most interventions were multicomponent (10/12), commonly integrating health education, exercise, psychosocial support, and self-care strategies. Nine programs combined face-to-face care with remote follow-up, and ten were directly led and delivered by nurses. Intervention duration ranged from four weeks to six months. Outcomes included functional, physiological, psychological, self-care, and quality-of-life domains. Favourable findings were frequently reported for functional capacity, anxiety and depression, self-care, self-efficacy, and health-related quality of life, whereas cardiovascular and physiological findings were less consistent. **Conclusions**: Nursing interventions for older adults with CHD are predominantly multicomponent and delivered across hospital and home settings. Further research should assess long-term outcomes and improve representation of very old, frail, and digitally vulnerable populations.

## 1. Introduction

Despite advances in the diagnosis and treatment of acute coronary syndromes [1], coronary heart disease (CHD) remains a major challenge for healthcare systems. It is estimated to affect approximately 240 million people worldwide and is a leading cause of morbidity, mortality and disability [2]. Its impact is particularly significant among older adults (people aged 60 years or over) [3,4]. In Spain, CHD makes a substantial contribution to the burden of disability among people aged over 70 years [5,6]. CHD is associated with loss of independence and poorer quality of life among older people living in the community [7,8]. The consequences of CHD extend beyond those affected, potentially increasing the need for family support and the complexity of care, thus resulting in physical, emotional and organisational implications for caregivers [9]. At the same time, CHD management has progressively shifted from hospital to outpatient and community settings, requiring greater continuity and coordination across different levels of care [10].

CHD encompasses the clinical manifestations resulting from reduced blood and oxygen supply to the myocardium [11]. It is commonly associated with structural or functional abnormalities of the coronary circulation system and may present as angina pectoris, myocardial ischaemia, acute coronary syndrome or myocardial infarction [12]. Although therapeutic advances have improved prognosis following acute coronary events, many older people continue to experience physical, functional and psychosocial consequences that affect recovery and disease progression [1,13]. From a physical and functional perspective, CHD is associated with reduced functional capacity, loss of muscle mass and strength, limitations in activities of daily living and an increased risk of dependency [14,15,16,17]. These limitations may coexist with persistent fatigue, anxiety, depressive symptoms and fear of further cardiovascular events [18,19]. Collectively, these problems can increase psychological distress, hinder adjustment to the condition and impair well-being and quality of life [20,21]. These consequences also extend to the social domain, limiting participation in everyday activities and increasing demands for care and support from families and healthcare services [18,19,20,21]. In addition, the frequent coexistence of multimorbidity and frailty increases the use of healthcare services, the risk of hospitalisation and the costs associated with caring for this population [22,23]. These circumstances reinforce the need for continuous, integrated models of care that coordinate clinical management, functional recovery and psychosocial support across different levels and services within the healthcare system [24].

Cardiac rehabilitation is an essential component of secondary prevention and is recommended by major clinical practice guidelines for people with CHD [24]. However, functional recovery and adjustment to the condition often require ongoing clinical support [25]. Furthermore, the rates of participation, adherence and completion of cardiac rehabilitation remain low among older adults [26,27]. Nurses often coordinate these programmes and are responsible for key components such as education, self-care support and follow-up after discharge, helping to promote continuity of care and prevent or limit CHD-related functional decline [28,29,30]. However, rehabilitation programmes are usually evaluated as a whole package, and the contribution of nurses is rarely described [10,30]. Where it is reported, the evidence is highly heterogeneous in terms of intervention structure, duration, mode of delivery, care setting and level of nursing involvement [31]. Moreover, the oldest old people and those living with frailty remain under-represented [10,32,33]. Although a recent systematic review examined nurse-led consultations for secondary prevention in adults with CHD, it did not specifically address rehabilitation and functional improvement in older people or the broader range of interventions delivered across different care settings [10]. Therefore, the purpose of this scoping review was to examine and map nursing interventions implemented and evaluated to support the rehabilitation of and functional improvement in older adults with coronary heart disease, with particular attention to how these interventions are organised, delivered and integrated across healthcare settings. The specific objectives were to: (1) identify nursing interventions aimed at the rehabilitation of and functional improvement in older adults with CHD; (2) describe their organisational and delivery characteristics, including structure, components, duration, frequency, mode of delivery and healthcare setting; (3) characterise nurses’ roles and their level of involvement or leadership in the interventions; (4) identify the main health problems and functional domains addressed; (5) map the outcome domains and measurement instruments used in the included studies; and (6) identify key evidence gaps related to the delivery and implementation of these interventions.

## 2. Materials and Methods

### 2.1. Design

A scoping review was conducted in accordance with the methodological recommendations of the Joanna Briggs Institute (JBI) [34,35]. This review was reported in accordance with the PRISMA-ScR (Preferred Reporting Items for Systematic Reviews and Meta-Analyses Extension for Scoping Reviews) checklist [36]. The scoping review protocol was retrospectively registered with the Open Science Framework (OSF) on 27 August 2026, following completion of this review (DOI: https://doi.org/10.17605/OSF.IO/HC4KR). Consequently, although the protocol was developed prospectively, its registration does not constitute prospective documentation of the review methods.

### 2.2. Search Strategy

A comprehensive search was conducted between November 2025 and February 2026. The search strategy was developed following the three-step approach recommended by JBI for scoping reviews [35]. In the first step, an exploratory search was conducted in PubMed, Cochrane, CINAHL, Web of Science and Scopus to identify the natural-language terms and MeSH descriptors most relevant to the research question. The main natural-language terms included “older adults”, “coronary heart disease”, “nursing”, “intervention”, “functional rehabilitation” and their respective variations (see Appendix A.1). In the second step, these terms were combined with MeSH descriptors, including “Coronary Disease”, “Aged”, “Nurses”, “Therapeutics” and “Rehabilitation”, using the Boolean operators OR, AND and NOT. The NOT operator was used to exclude studies focused exclusively on pharmacological interventions. Finally, in the third step, the search strategy was adapted to the specific syntax of each database. The combination of natural-language terms and MeSH descriptors resulted in the following main search strategy, used in PubMed: ((((((((((Chronic ischemic heart disease[Title/Abstract]) OR (Coronary heart disease[Title/Abstract])) OR (Acute coronary syndrome[Title/Abstract])) OR (Post-heart attack[Title/Abstract])) OR (Myocardial ischemia[Title/Abstract])) OR (coronary disease[MeSH Terms])) OR (Myocardial ischemia[MeSH Terms])) AND (((((((Elderly[Title/Abstract]) OR (Aged[Title/Abstract])) OR (Senior adults[Title/Abstract])) OR (Older adults[Title/Abstract])) OR (Older people[Title/Abstract])) OR (Aging[Title/Abstract])) OR (aged[MeSH Terms])))) AND ((((((((((Intervention[Title/Abstract]) OR (Program[Title/Abstract])) OR (Treatment[Title/Abstract])) OR (Therapy[Title/Abstract])) OR (Strategy[Title/Abstract])) OR (Method[Title/Abstract])) OR (Care[Title/Abstract]))) OR (therapeutics[MeSH Terms]))) AND ((((nurses[MeSH Terms]) OR (nurse*[Title/Abstract])) OR (“nurse interventions”[Title/Abstract])) OR (“nurse programs”[Title/Abstract])) AND (((((((((((((Functional rehabilitation[Title/Abstract]) OR (Physical rehabilitation[Title/Abstract])) OR (Cardiac rehabilitation[Title/Abstract])) OR (Comprehensive rehabilitation[Title/Abstract])) OR (Motor rehabilitation[Title/Abstract])) OR (Functional recovery[Title/Abstract])) OR (Functional capacity[Title/Abstract])) OR (Functional mobility[Title/Abstract])) OR (Activities of daily living[Title/Abstract])) OR (Quality of life[Title/Abstract])) OR (Community-based rehabilitation[Title/Abstract])) OR (Post-hospital rehabilitation[Title/Abstract])) OR (rehabilitation[MeSH Terms]))) NOT ((“Drug Therapy”[MeSH Terms] OR “Pharmacological treatment”[Title/Abstract] OR “Medication”[Title/Abstract] OR “Pharmacotherapy”[Title/Abstract])). This core search string served as the basis for the other databases, with the corresponding formatting modifications applied to each one (see Appendix A.3).

### 2.3. Eligibility Criteria

In line with the methodological guidance of the Joanna Briggs Institute (JBI) for conducting scoping reviews, the PCC framework (P: participants; C: concept; C: context) was used to define the inclusion criteria [37].

#### 2.3.1. Type of Participants

This scoping review considered all studies focusing on older adults with CHD. The initial eligibility criteria specified that studies should have a mean participant age of 60 years or older and include patients diagnosed with coronary heart disease or any of its clinical manifestations or complications. Eligibility was assessed at the study level, based on the characteristics of the study population, rather than at the individual participant level.

For studies including participants of different ages or with different diagnoses, the initial eligibility criteria required that data relating specifically to older adults with coronary heart disease could be identified separately. However, during study selection, three studies reporting mean participant ages below 60 years were retained despite not strictly meeting the original age criterion. Their reported age distributions indicated that participants aged 60 years or older were included, although age-specific outcome data could not be extracted separately. This decision therefore represented a deviation from the initially specified eligibility criteria and it has been acknowledged as a study limitation.

#### 2.3.2. Concepts

This review considered studies focused on nursing interventions implemented and evaluated to support rehabilitation and functional improvement in older adults with coronary heart disease. Non-pharmacological rehabilitation programmes and models of clinical care led or coordinated by nurses were included. Multidisciplinary interventions were also considered when nurses’ involvement and roles were clearly described. Studies in which the nursing role could not be identified from the description of the intervention were excluded at the full-text stage. Furthermore, interventions had to have been implemented directly with patients. They could include health education, psychosocial or behavioural support, promotion of self-care, clinical follow-up, care coordination and other strategies aimed at maintaining or improving functional capacity and independence. Studies assessing domains related to rehabilitation and functional improvement were also considered. These domains included physical and functional capacity, independence, quality of life, self-efficacy and other biopsychosocial outcomes. These outcomes were included to identify which dimensions had been assessed, rather than to compare the effectiveness of the interventions.

Studies focusing exclusively on pharmacological interventions were excluded. Studies describing theoretical programmes, protocols or proposed interventions that had not been implemented with patients were also excluded.

#### 2.3.3. Context

This review considered interventions delivered in any healthcare or community setting related to the care and rehabilitation of older adults with CHD. This included hospitals, cardiac rehabilitation units, outpatient clinics, primary care, community services and home-based care. Interventions delivered through digital or telehealth modalities were also included. No restrictions were applied according to level of care, provided that the nursing intervention and its mode of delivery were clearly described.

#### 2.3.4. Type of Sources

This scoping review considered quantitative and mixed-method studies. Eligible quantitative designs included randomised controlled trials, quasi-experimental studies and pre-test–post-test studies evaluating an implemented intervention. Mixed-method studies were included when they incorporated a quantitative or qualitative evaluation of the implementation, delivery or outcomes of the intervention. Studies published in English or Spanish from 1 January 2020 to the date on which the search was conducted were considered eligible. The publication date restriction was established to focus this review on recent nursing interventions and contemporary approaches to cardiac rehabilitation, including developments in care delivery, continuity of care, and digital health technologies. This timeframe was selected to provide a focused mapping of recent evidence rather than a comprehensive historical synthesis. Nevertheless, relevant studies published before 2020, including those addressing home-based cardiac rehabilitation, may have been excluded as a result of this restriction. Literature reviews, protocols, editorials, letters to the editor and other documents that did not report data derived from an implemented intervention were excluded.

### 2.4. Study Selection

Records retrieved from five databases (Web of Science, CINAHL, Scopus, PubMed and Cochrane) were exported and managed for screening. Duplicate records were first removed, and a date filter was applied to restrict the evidence to studies published between 2020 and 2026. The remaining records were then screened sequentially and independently by two reviewers. Records that were not intervention studies were excluded. The titles and abstracts of the remaining records were subsequently screened, and those that did not meet the criteria relating to the population, concept or review objective were excluded. Full-text articles were then assessed against the predefined eligibility criteria. Any disagreements between the reviewers were resolved through discussion and consensus.

### 2.5. Data Extraction and Synthesis

Data were extracted by one reviewer using a standardised data-charting instrument specifically developed for this review. A second reviewer systematically verified the extracted data for all included studies against the original publications, checking the accuracy and completeness of each data field. Any discrepancies identified during verification were discussed between the two reviewers and resolved through reaching a consensus, with the agreed corrections incorporated directly into the data-charting instrument. The instrument was developed in accordance with methodological guidance from the Joanna Briggs Institute and aligned with the review objective and research questions. Before final use, the instrument was piloted on a sample of the included studies and refined as necessary.

The following data were charted: authorship, year and country of publication; study design and objective; population characteristics, including sample size and mean age; type and domain of the nursing intervention; healthcare setting and mode of delivery; professionals involved and nurses’ roles; intervention structure, components, duration, frequency and follow-up; functional and biopsychosocial domains assessed; measurement instruments used; and the main findings reported by the studies. Any uncertainties or discrepancies arising during data extraction were resolved through discussion and reaching a consensus between the two reviewers. Intervention characteristics and outcomes were then grouped into categories and summarised as frequencies. Outcomes were charted as reported by the original authors. Given the diversity of instruments, scoring ranges and assessment time points, outcome data were not harmonised or pooled, and the results were synthesised descriptively by outcome domain.

### 2.6. Quality Assessment

The methodological quality and risk of bias of the included studies were assessed using the JBI Critical Appraisal tools specific to each study design [38,39]. The mixed-method study was assessed using the Mixed Methods Appraisal Tool (MMAT) [40]. Studies were grouped according to their methodological design, and the appraisal results were recorded in design-specific tables. The columns in each table corresponded to the criteria established by the relevant appraisal tool. This process yielded an individual quality score for each study, enabling the main methodological strengths and limitations to be identified and supporting a critical interpretation of the included evidence (see Appendix B).

## 3. Results

### 3.1. Search Results and Study Selection Process

**Figure 1:**
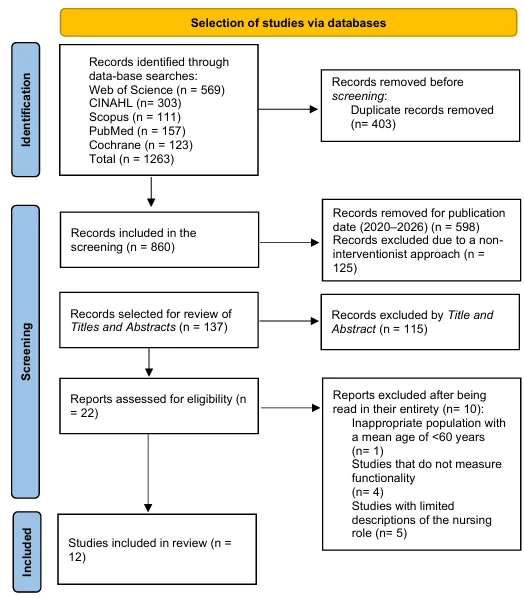
PRISMA-ScR flow diagram of the study selection process.

During the initial identification stage, records were retrieved from Web of Science (*n* = 569), CINAHL (*n* = 303), Scopus (*n* = 111), PubMed (*n* = 157) and Cochrane (*n* = 123), yielding a total of 1263 records. After removal of 403 duplicates, 860 records remained. A date filter was then applied to restrict the evidence to the previous six years, resulting in the exclusion of 598 records and reducing the sample to 262 articles. Screening for an intervention focus further reduced the number of records to 137. Title and abstract screening led to the exclusion of an additional 115 records, leaving 22 articles for full-text assessment. Of these, ten did not meet the eligibility criteria and were excluded. Lastly, 12 studies were included in this review. The study selection process is presented in the PRISMA-ScR flow diagram (Figure 1).

### 3.2. Characteristics of the Studies and Study Population

Twelve primary studies were included, comprising randomised controlled trials (*n* = 9), quasi-experimental studies (*n* = 2) and a mixed-method study (*n* = 1). Most studies were conducted in China (*n* = 8), followed by Iran (*n* = 2), Jordan (*n* = 1) and Denmark (*n* = 1). The combined sample comprised 1345 participants, with individual study sample sizes ranging from 30 to 200 patients. Most included studies reported a mean participant age of 60 years or older [41,42,43,44,45,46,47,48,49]. However, three studies reported mean ages below this threshold [50,51,52]. These studies were retained despite not strictly meeting the initially specified mean-age criterion, representing a deviation from the original eligibility criteria. Their reported age distributions suggested that older adults were represented within the study populations; however, the available aggregate data did not allow the proportion of participants aged 60 years or older to be determined. Eligibility was assessed at the study level rather than at the individual participant level. This deviation should be considered when interpreting the applicability of the findings specifically to older adults. All participants had serious cardiac conditions, primarily coronary heart disease, acute coronary syndrome and acute myocardial infarction. Alongside the primary cardiac diagnosis, the most common comorbidities were hypertension, diabetes mellitus and dyslipidaemia (see Table 1).

### 3.3. Characteristics, Organisation and Delivery of Nursing Interventions

Table 2 summarises the included interventions by clinical population, mean age, duration, care setting, mode of delivery, nursing role and outcome domains assessed.

#### 3.3.1. Structure and Components of the Interventions

Most programmes combined several therapeutic components. Ten of the twelve studies incorporated two or more components within a single care pathway, typically education, exercise and emotional support [41,42,43,44,45,46,47,50,51,52]. Only two studies were structured around a single core component: peer support provided by patients with previous experience of the condition [48,49].

Health education was the most frequently reported component and was included in eleven programmes [41,42,43,44,45,46,47,49,50,51,52]. The format varied considerably across studies. Li et al. [47] showed brief five-to-eight-minute videos in patients’ rooms during their hospital stay. Hao et al. [44] delivered multimedia materials daily via a messaging application. Su & Yu [51] integrated educational content into a web-based platform that could be accessed from home. Cao et al. [41] provided individual face-to-face sessions supplemented by manuals, leaflets and self-monitoring diaries, while Heidari et al. [45] combined face-to-face, virtual and telephone sessions with an educational leaflet. The content was broadly similar across studies and included information about the condition, medication management, dietary advice, physical activity and recognition of warning signs.

Physical training was incorporated with varying degrees of structure. Four programmes prescribed a structured and progressive exercise load [42,43,44,47]. Hao et al. [44] progressed participants from basic mobilisation in bed to walking and squats, adjusting the level of exertion according to tolerance. Li et al. [47] instructed participants to regulate exercise intensity using the Borg scale and to record it in a diary. Duan and Ren [42] developed individualised rehabilitation programmes with a gradual increase in physical activity under nurses’ supervision. Guo et al. [43] incorporated physical training into a standardised cardiac rehabilitation protocol. A further four programmes promoted physical activity without prescribing a specific exercise load [45,50,51,52]. Su & Yu [51] used goal setting and self-monitoring with pedometers. Snaid and Qibalat [50], Heidari et al. [45] and Zou et al. [52] incorporated physical activity as an educational component and monitored it during follow-up.

Psychological and behavioural support was included in seven programmes [41,43,44,46,48,50,52]. Strategies included relaxation, cognitive restructuring, mindfulness, emotional regulation, stress coping, self-motivation and empathetic support. Some programmes also involved family members or peer mentors with previous experience of the condition [44,48].

Promotion of self-care was included in eight programmes [41,42,44,45,46,47,49,51]. This was implemented through goal setting agreed jointly with patients [41,42,46,51] and systematic recording of daily activities in diaries [41,47]. Su & Yu [51] also incorporated a visual feedback system that enabled patients to track their own progress using a traffic-light display of goal attainment. Heidari et al. [45] tailored self-care support to the deficits identified in each patient.

Three programmes explicitly incorporated peer support, although using different approaches [48,49,51]. Roshandel et al. [49] assigned the delivery of the educational content to trained peer patients, who shared videos via a messaging application. Su & Yu [51] created professionally moderated messaging groups, preceded by a face-to-face orientation session. Pedersen et al. [48] used one-to-one matching between mentors and patients, with flexible contact tailored to each participant, during which mentors shared their own recovery experiences to encourage continued engagement with rehabilitation.

#### 3.3.2. Care Settings and Modes of Delivery

The interventions were implemented at different points along the care pathway. The most common model involved continuity from hospital to home and was used in six programmes [41,42,47,50,51,52]. In these cases, the intervention began during hospital admission and continued after discharge through home-based follow-up. Three programmes were delivered exclusively at home following hospital discharge [46,48,49]. Two programmes initiated the intervention during hospital admission [43,45], one of them in an intensive care unit following acute myocardial infarction [45], although the articles did not specify whether the intervention continued after discharge. Only one programme was delivered in a community setting [44].

Regarding mode of delivery, nine programmes used a blended approach combining face-to-face interventions with one or more forms of remote follow-up [41,42,43,44,45,47,48,50,52]. Two studies relied predominantly on digital tools [49,51], while one was delivered exclusively by telephone [46]. Messaging applications and web-based platforms were among the channels used for remote follow-up [44,49,51,52]. Scheduled telephone follow-up was used in five programmes [41,46,48,50,52] and was mentioned without specifying frequency in a further two [43,45]. Face-to-face home visits were explicitly described in two programmes [44,48] and mentioned without further detail in a third study [43]. Two other programmes used audiovisual materials without internet connectivity or remote contact [42,47], while another study used a pedometer for self-monitoring [51].

#### 3.3.3. Duration, Frequency and Intensity

The duration of the programmes ranged from four weeks to six months. The most common timeframe was 12 weeks, adopted by five studies [41,42,44,47,51]. Five programmes were delivered over periods of eight weeks or less [43,45,46,49,52], while the longest interventions lasted six months [48,50].

The frequency of contact varied across programmes. Four programmes included components delivered daily [43,44,47,52], while others combined weekly sessions with more frequent activities [43,46,50,52]. In some cases, contact frequency decreased progressively over the course of the intervention [41,44]. Other programmes used a flexible schedule according to patient needs [48] or allowed for contact on demand through a digital platform [51].

Where specified, individual sessions generally lasted between 20 and 60 min. The shortest formats involved audiovisual content and self-motivation exercises [43,47]. Face-to-face sessions tended to be longer, lasting 30–45 min [45], at least one hour during the hospital phase [41], and 30–60 min for psychological counselling [43]. Snaid and Qibalat [50] set cognitive behavioural therapy sessions at 30 min. Four studies did not specify session duration [42,49,51,52].

Follow-up periods after completion of the intervention ranged from one to six months. Six studies assessed outcomes at two or more time points [41,45,47,48,51,52].

### 3.4. Nursing Role and Level of Leadership

Nurses’ involvement was identified in all included studies, although the degree of leadership varied. Two levels of involvement were identified: direct delivery of the intervention by nurses (*n* = 10) and coordination and supervision of other providers who delivered it (*n* = 2) [48,49].

Ten programmes were led and delivered directly by nurses [41,42,43,44,45,46,47,50,51,52]. Nurses conducted the initial assessment, set goals, delivered the intervention content and provided follow-up. The level of specialist expertise required varied across programmes. Guo et al. [43] involved mental health nurses working in coordination with cardiac rehabilitation nurses. Su & Yu [51] assigned the programme to a nurse with experience in cardiovascular nursing. Hao et al. [44] included specialist nurses within a cardiac disease management team, where they took the lead in delivering the programme. In the two remaining programmes, nurses trained and supervised peer mentors or peer patients, who delivered the intervention content, while nurses retained responsibility for coordinating the programme [48,49].

Some studies provided details of the nurses’ qualifications, experience and responsibilities [43,44,51]. In others, their involvement was inferred from the intervention content or its grounding in nursing models rather than being explicitly stated [41,52].

### 3.5. Health Problems and Domains Assessed

Most articles focused on older adults with a general diagnosis of coronary heart disease [41,42,44,47,48,49,51]. The remaining studies focused on populations defined by a specific condition, event or procedure: patients following acute coronary syndrome [52], patients with stable angina [50], patients admitted to an intensive care unit after acute myocardial infarction [45], or patients recovering from percutaneous coronary intervention [43,46]. The most common comorbidities included hypertension, diabetes mellitus and dyslipidaemia [42,43,44,51]. Symptoms of anxiety and depression were also identified [52], and one study included type D personality as an eligibility criterion [41].

The included interventions addressed different dimensions of care for older adults with CHD, which can be grouped into four broad domains: (1) biological and functional; (2) psychological and emotional; (3) social, educational and self-care; and (4) healthcare utilisation and patient experience.

### 3.6. Outcomes Assessed

#### 3.6.1. Biological and Functional Outcomes

##### Functional and Exercise Capacity

Several programmes (*n* = 4) assessed patients’ functional capacity using the six-minute walk test, with improvements observed in the intervention group in all cases [42,44,47,50]. Improvements were reported by Li et al. [47] (495.55 m vs. 370.24 m; *p* < 0.001) and Hao et al. [44] (326.82 m vs. 273.24 m; *p* < 0.001). Aerobic capacity was assessed in only one study, which also reported results favouring the intervention [42] (*p* < 0.01).

##### Cardiovascular and Physiological Parameters

Several studies assessed participants’ clinical parameters (*n* = 4). Duan and Ren [42] reported a higher left ventricular ejection fraction in the intervention group (38.3% vs. 35.7%; *p* < 0.001), while Snaid and Qibalat [50] reported a lower Tei index at six months (0.42 ± 0.30 vs. 0.54 ± 0.32), with statistical significance reported for the magnitude of the reduction (0.12 vs. 0.03; *p* = 0.041). Zou et al. [52] observed a reduction in systolic blood pressure at 12 weeks (β = −3.32; *p* = 0.01), with no differences in diastolic blood pressure, body mass index, lipid levels, blood glucose or smoking status. Su & Yu [51] found no significant differences in blood pressure, body mass index or waist circumference.

#### 3.6.2. Psychological and Emotional

##### Anxiety and Depression

Six studies assessed symptoms of anxiety and depression. Snaid and Qibalat [50] reported lower anxiety (49.24 ± 25.22 vs. 67.56 ± 28.64) and depression scores (49.24 ± 28.54 vs. 60.37 ± 30.22) at six months, with significantly greater mean reductions in the intervention group (anxiety, *p* = 0.016; depression, *p* = 0.029). Guo et al. [43] documented favourable differences in anxiety (25.71 vs. 27.95; *p* = 0.013) and depression (30.98 vs. 34.30; *p* < 0.001). Cao et al. [41] reported lower levels at one and three months (anxiety, β = −1.84, 95% CI −2.91 to −0.77; depression, β = −1.41, 95% CI −2.27 to −0.55; *p* < 0.05). Zou et al. [52] observed sustained reductions in depression (β = −2.01 at T1 and −2.08 at T2; *p* < 0.001) and anxiety (β = −1.02 at T1, *p* < 0.001; β = −0.93 at T2, *p* = 0.002). By contrast, Su & Yu [51] and Pedersen et al. [48] found no statistically significant improvements.

##### Kinesiophobia, Stress, Resilience and Mood

Some authors assessed specific coping-related variables (*n* = 4). Guo et al. [43] reported lower kinesiophobia scores at two months (33.88 vs. 41.32; *p* < 0.001), indicating less fear of movement. Zou et al. [52] observed a reduction in perceived stress (β = −1.18 at T1, *p* < 0.001; β = −1.26 at T2, *p* = 0.001). Hua et al. [46] found greater resilience (65.79 ± 13.40 vs. 58.74 ± 12.77; *p* < 0.05). Hao et al. [44] reported lower scores for negative emotions (13.28 vs. 19.87) and higher scores for hope (37.99 vs. 32.83; *p* < 0.001).

#### 3.6.3. Social, Educational and Self-Care Outcomes

##### Self-Care Skills and Adherence

Several studies assessed self-care competencies and health behaviours (*n* = 8). Duan and Ren [42] observed higher self-care capacity scores (144.5 vs. 137.1; *p* < 0.001), while Hao et al. [44] reported improvements across all dimensions of self-care (*p* < 0.001). Heidari et al. [45] reported substantial differences in self-care skills (102.10 vs. 53.03), readiness (31.60 vs. 17.46) and knowledge (112.77 vs. 41.74) at eight weeks (*p* < 0.005). Li et al. [47] reported higher self-management scores (3.84 vs. 2.78; *p* < 0.001), a greater proportion of participants exercising weekly (85% vs. 31%; *p* < 0.001) and a lower rate of smoking relapse (6% vs. 43%; *p* = 0.028). Roshandel et al. [49] reported increases in self-care (18.46 ± 7.93 vs. 7.06 ± 3.71) and treatment adherence (10.93 ± 4.75 vs. 3.83 ± 1.55; *p* < 0.001). Su & Yu [51] documented better health-promoting behaviours (110.35 vs. 95.00; *p* < 0.001), a higher daily step count (9502 vs. 7298; *p* = 0.006) and less sedentary time (1240 vs. 2011 min/week; *p* = 0.004). Pedersen et al. [48] found no significant differences in diet or physical activity. Zou et al. [52] reported improvements in dietary behaviour (β = +0.069; *p* = 0.04) and physical activity levels (β = +177.542; *p* = 0.01) at 12 weeks.

##### Self-Efficacy

Four studies assessed self-efficacy using different instruments, with inconsistent findings. Su & Yu [51] reported higher scores (3.31 vs. 2.79; *p* = 0.005), Guo et al. [43] found greater exercise self-efficacy (52.90 vs. 44.86; *p* = 0.009), and Cao et al. [41] reported an increase at three months (β = +2.08; *p* < 0.05). Pedersen et al. [48] found no significant differences.

##### Health-Related Quality of Life

The vast majority of studies (*n* = 11) assessed quality of life. Guo et al. [43] reported higher scores (85.43 vs. 79.21; *p* < 0.001), as did Hua et al. [46] (67.06 ± 14.83 vs. 60.76 ± 11.13; *p* = 0.029), Snaid and Qibalat [50] (*p* < 0.001) and Roshandel et al. [49] (8.20 ± 4.69 vs. 2.73 ± 1.62; *p* < 0.001). Heidari et al. [45] reported a substantial difference, with scores increasing from 58.94 to 168.43 in the intervention group compared with 54.69 to 72.00 in the control group (*p* < 0.001). Duan and Ren [42] (31.4 vs. 29.8) and Hao et al. [44] (127.84 vs. 103.69) also reported significant improvements in quality of life (*p* < 0.001). Cao et al. [41] observed greater angina stability (β = +20.98 at one month and +29.23 at three months; *p* < 0.05). Zou et al. [52] reported improvements in the physical (β = +0.088; *p* = 0.03) and emotional (β = +0.294; *p* < 0.001) subscales and in the overall score (β = +0.147; *p* < 0.001) immediately after the intervention, although at 12 weeks, the effect was maintained only for the emotional subscale (β = +0.249; *p* = 0.001). Su & Yu [51] found no statistically significant difference in the overall MacNew score (5.90 vs. 5.58; β = 0.40; 95% CI −0.01 to 0.80; *p* = 0.057), although significant improvements were observed in the physical (β = 0.45; *p* = 0.049) and social (β = 0.54; *p* = 0.025) subscales. Pedersen et al. [48] found no significant quantitative differences; however, in the qualitative component, participants reported that mentors’ empathetic support reduced their fear of death and feelings of loneliness during the early period following hospital discharge.

#### 3.6.4. Use and Experience of the Service Outcomes

##### Healthcare Utilisation

Su & Yu [51] found no relevant between-group differences in unplanned healthcare use. Two participants in the intervention group were readmitted for chest pain, one of whom underwent percutaneous coronary intervention, while in the control group, two participants attended the emergency department for cardiac symptoms and a further two were readmitted for revascularisation. Survival analysis showed a slightly longer time to hospital admission in the intervention group, although this did not reach statistical significance (*p* = 0.25). Hao et al. [44] reported greater adherence to scheduled clinical follow-up visits in the intervention group (79.1% vs. 60.4%; *p* < 0.05).

##### Accessibility and Programme Retention

Pedersen et al. [48] observed a higher proportion of participants being offered community-based rehabilitation in the intervention group (48% vs. 34%), while only the control group received an exclusively hospital-based rehabilitation offer (9%). Zou et al. [52] reported an intervention completion rate of 76% and a lower dropout rate in the intervention group (15% vs. 21%). Completion of a greater number of sessions was associated with a greater reduction in depressive symptoms (r = 0.324; *p* = 0.003). Other studies reported losses to follow-up without analysing them as an outcome [41,47,51].

##### Satisfaction and Acceptability

Zou et al. [52] reported high acceptability of the intervention, with positive responses ranging from 93% to 100% among participants who completed the questionnaire. However, 15% reported experiencing difficulties during the programme. No adverse events were recorded. Pedersen et al. [48] reported that 34% of participants were satisfied and 58% were very satisfied with the intervention, while 42% perceived some benefit and 32% perceived a substantial benefit. Cao et al. [41] found a significant improvement in treatment satisfaction at three months (β = +6.95; *p* < 0.05).

### 3.7. Results of the Quality Assessment

The methodological quality of the included studies was assessed using the Joanna Briggs Institute’s (JBI) critical appraisal tools, according to study design [38,39], and the Mixed Methods Appraisal Tool (MMAT) [40]: randomised controlled trials (RCTs), quasi-experimental studies and mixed-method studies.

The randomised controlled trials showed moderate-to-good methodological quality, with scores ranging from 9 to 11 out of 13 items. Four studies scored 11 points [46,47,51,52], one scored 10 [41], and four scored 9 [42,43,49,50]. The main methodological limitations identified were the absence of blinding of participants and intervention providers, together with limited clarity regarding allocation concealment. In educational or behavioural interventions, blinding is often difficult to implement; therefore, its absence should be interpreted as an inherent limitation of the study design rather than as a direct indicator of poor methodological quality (Appendix B.1).

The two quasi-experimental studies showed high methodological quality, each scoring 8 out of 9 items [44,45]. As specific limitations, Hao et al. [44] did not include multiple measurements before and after the intervention. Heidari et al. [45] did not adequately describe follow-up procedures or losses to follow-up. In the latter study, discrepancies were also identified between the figures reported in the abstract and those presented in the result tables, as well as in the quality-of-life values, whose magnitude was difficult to interpret in relation to the usual range of the instrument used (see Appendix B.2).

The mixed-method study showed high methodological quality, scoring 5 out of 5 items on the MMAT. It provided an appropriate justification for the study design, integrated the quantitative and qualitative components, and presented a coherent interpretation of the findings (see Appendix B.3).

Overall, the critical appraisal identified several methodological strengths across the included studies while also highlighting limitations related to blinding, allocation concealment, and follow-up procedures. Although most studies fulfilled the majority of the appraisal criteria, these findings should not be interpreted as evidence of intervention effectiveness or as indicating a high level of certainty in the overall body of evidence. The appraisal results were used to contextualise the findings reported by individual studies and identify methodological limitations relevant to their interpretation.

## 4. Discussion

The aim of this scoping review was to examine and map the nursing interventions implemented and evaluated to support the rehabilitation of and functional improvement in older adults with coronary heart disease. Our findings indicate that most nursing interventions extended beyond hospital discharge, highlighting the importance of continuity of care during the transition from hospital to home. However, access to these services may be constrained by eligibility criteria and technological requirements, potentially limiting participation among patients who face the greatest barriers to accessing care. In this context, older adults with CHD are particularly vulnerable to these barriers due to the interaction of multiple factors. The high burden of age-related comorbidity [14,20] is compounded by the frequent presence of anxiety and depressive symptoms following a cardiac event [18,19], together with multidimensional frailty that affects quality of life [21]. Considerable heterogeneity was also observed in the design and components of the programmes analysed, consistent with previous reviews reporting substantial diversity among nurse-led cardiac rehabilitation models [31,53,54].

The interventions were delivered across different stages of the care pathway, although they were concentrated particularly in the post-discharge period. This pattern is consistent with the importance attributed to continuity of care in coronary heart disease [55] and with the favourable outcomes reported for home-based transitional care models in older adults [56]. Okazaki et al. [57] documented a similar pattern regarding heart failure, where structured nursing interventions support self-care after discharge. However, this continuity does not appear to be fully integrated into the organisation of healthcare. As highlighted by Korkmaz Yaylagul et al. [58], the sustainability of these programmes depends not only on their effectiveness, but also on organisational models capable of embedding them into clinical practice.

The programmes differed in the degree of structure applied to physical activity interventions. Some incorporated progressive exercise prescriptions and monitoring of exercise intensity [42,44,47], whereas others focused on physical activity promotion, education, and follow-up without specifying a progressive exercise load [45,50,51,52]. Improvements in functional outcomes were reported in some studies incorporating structured exercise components [42,44,47]. However, differences in study populations, intervention components, outcome measures, assessment time points, and study designs preclude direct comparisons of effectiveness between these approaches. Previous evidence has highlighted the role of structured and progressive exercise in addressing physical deconditioning associated with ageing and disease [59,60]. Within the interventions mapped in this review, the nursing role extended beyond education and support to include the prescription, progression, and monitoring of rehabilitation activities.

However, the way in which nurses exercised leadership varied across interventions, while maintaining their central coordinating role. In some cases, this included delegating specific intervention components to expert patients trained by the research team. Beyond its potential to optimise the use of healthcare resources, this peer-led approach may also strengthen the intervention by drawing on shared experience and providing support that is accessible and relatable to participants. Peer mentoring has been shown to improve health literacy and self-management capacity [61]. By promoting a person-centred model of care, these interventions can help older adults feel more capable and engaged, encouraging more active and independent management of their health [62,63]. This effect appears to differ from that of unstructured social support, whose benefits have been associated primarily with emotional relief rather than necessarily with behavioural change [64]. These findings highlight the diversity of supportive and structured components incorporated into nursing interventions, although the relative contribution of each component remains unclear. Despite existing evidence on structured nursing interventions in patients with multimorbidity and their implications for healthcare resource use [65,66], none of the studies included in this review reported the workload associated with programme delivery, the patient-to-professional ratio, or the training required for replication. This lack of information limits the assessment of the feasibility and reproducibility of these interventions in routine clinical practice.

As an alternative to face-to-face contact, some programmes used general-purpose messaging applications and a web-based platform to maintain follow-up after discharge. Digital platforms enabled continued follow-up without requiring patients to travel, a considerable advantage for those with limited mobility and consistent with findings reported in other chronic conditions [67,68,69]. However, digitalisation introduces an additional barrier to access by excluding patients without internet access, suitable devices or sufficient digital skills [51,52]. Thus, despite digitalisation potentially improving access for some patients, it may also create new inequalities, particularly among older people and those with lower levels of education. This issue has already been described in relation to technology adoption among older adults with chronic conditions [26], socioeconomic and geographical inequalities in geriatric telerehabilitation [70], and the under-representation of frail patients in digital health trials [71]. In routine clinical practice, this suggests that nurses should avoid applying a standard rehabilitation protocol. Instead, they should conduct a comprehensive initial assessment to identify the specific barriers faced by each older adult and select the rehabilitation modality best suited to their needs and available resources.

Alongside barriers to access, the findings also support psychosocial intervention as a priority for addressing isolation and kinesiophobia, both of which can negatively affect adherence to physical rehabilitation [72]. Sakamoto et al. [19] further support this association by linking depressive symptoms with poorer physical performance during rehabilitation. This pattern extends beyond cardiology, as seen in recovery after hip fracture, where fear of falling can hinder functional recovery if it is not addressed alongside motor impairment [73,74]. Fear of movement therefore represents an important barrier to rehabilitation, reinforcing the need to incorporate emotional assessment before prescribing exercise [75,76].

The duration and persistence of intervention effects remain underexplored. Although six studies assessed outcomes at two or more time points, only Zou et al. [52] explicitly examined whether the effects were sustained over time. Improvements in the physical and overall dimensions of quality of life diminished at 12 weeks, whereas the effect on the emotional dimension was maintained. Moreover, the association between the number of sessions completed and improvement in depressive symptoms suggests that adherence may influence intervention effectiveness. As no study included follow-up beyond six months, it remains unclear whether the benefits persist once professional support has ended. This reinforces one of the main gaps identified in the cardiac rehabilitation literature [32,33]. This uncertainty is compounded by considerable variation in the duration and frequency of contact across studies. In contrast to the shorter programmes described in younger adults, older adults may benefit from longer periods of adaptation and a gradual reduction in contact frequency to consolidate self-care [27,56,77,78,79]. This approach would allow more intensive support to be provided after discharge and gradually reduced as patients become more independent [57]. Comparisons of intervention duration are further complicated by the substantial heterogeneity of the instruments used to assess mental health, self-efficacy and quality of life. This variability, also reported in previous reviews of nurse-led cardiac rehabilitation [31,53], reflects the lack of standardisation in outcome assessment within this field. Consequently, although this review identifies common trends across interventions, the diversity of outcome measures limits the ability to determine the magnitude and consistency of their effects with precision.

Finally, the nursing role in these interventions should be interpreted with caution. In all included studies, the intervention was compared with usual care or standard rehabilitation, and none evaluated nurse involvement as an independent factor. Moreover, the nursing role was largely consistent across studies, with nurses delivering the intervention directly in ten of the twelve studies. The favourable findings reported should therefore be attributed to the interventions as a whole rather than to the nursing role itself.

The methodological appraisal provides additional context for interpreting the reported outcomes. Although the included studies generally met several quality criteria, limitations related to blinding, allocation concealment, and follow-up procedures were identified. Importantly, methodological quality at the individual study level does not establish the overall certainty of evidence regarding intervention effectiveness. Given the heterogeneity of interventions and outcome measures, together with the limited duration of follow-up, the favourable outcomes reported in individual studies should be interpreted cautiously. Further research with robust methodological designs, standardised outcome measures, and longer follow-up periods is needed to establish the consistency and sustainability of these findings.

### Limitations of This Study

This scoping review has several methodological limitations arising from both the review methodology and the characteristics of the included primary studies.

Regarding the scoping review methodology, a date restriction was applied, limiting the search to the previous five years. This methodological decision was based on the rapid technological evolution of digital health interventions, which reduces the current clinical applicability of earlier technological approaches. Although this restriction may have led to the omission of relevant earlier studies, the search was conducted across five databases and yielded twelve studies with a combined sample of 1345 patients. Additionally, three studies with mean participant ages below 60 years were retained despite not strictly meeting the original age criterion. Given that age-specific outcomes could not be extracted separately, their inclusion may limit the applicability of the findings to populations exclusively aged 60 years or older. In addition, during study selection, efforts were made to include interventions grounded in foundational nursing models, particularly those of Dorothea Orem and Virginia Henderson. This approach enabled this review to capture both contemporary nursing practice and the theoretical frameworks underpinning the discipline. Although the review protocol was developed before the review process began, its retrospective registration limits the independent verification of the methodological decisions established prospectively. Moreover, the requirement that the nursing role be explicitly described may have excluded programmes in which nurses took part but their role was not reported, which partly explains the small number of included studies. Given the variability, a scoping review design was considered appropriate for mapping the available evidence, and methodological quality was assessed using the Joanna Briggs Institute’s critical appraisal tools. However, although methodological quality was assessed at the individual study level, no formal assessment of the overall certainty of evidence was undertaken. Therefore, the appraisal findings should not be interpreted as establishing the effectiveness of nursing interventions, particularly given the limited follow-up periods and variability in the outcomes assessed.

Regarding the included primary studies, another limitation is the geographical concentration of studies in Asia. This geographical imbalance, together with the use of locally dominant platforms such as WeChat rather than those more commonly used in Europe, limits the direct transferability of the findings to other health and social care contexts, including our own. Future trials should therefore adapt, implement and validate these multicomponent interventions in Western populations. The substantial heterogeneity in programme duration and the measurement scales used should also be considered, as should the impossibility of blinding patients in educational interventions. This heterogeneity precludes meaningful comparisons of effectiveness between intervention approaches. Therefore, the findings should be interpreted as a descriptive mapping of intervention characteristics and reported outcomes, rather than as evidence of the greater effectiveness of particular rehabilitation strategies. In addition, routine reliance on technology may introduce selection bias by frequently excluding the oldest and frailest patients, as well as those without internet access; so, the findings should not be generalised to all older adults with CHD. Future nursing care plans should therefore adopt hybrid approaches that combine digital literacy support with traditional face-to-face follow-up to promote equitable access. Finally, there was a notable lack of reporting on long-term clinical outcomes, such as cardiovascular mortality or hospital readmission, as the literature focused predominantly on functional outcomes. Given that the ultimate aim is to improve both prognosis and quality of life, future research should prioritise follow-up periods exceeding 12 months and integrate the outcomes of nursing interventions directly into older adults’ clinical records.

## 5. Conclusions

Holistic, multicomponent interventions were associated with favourable findings for functional capacity and kinesiophobia. These approaches are characterised by the simultaneous integration of psychological and behavioural support, physical exercise and structured education. Other strategies based primarily on digital technologies (eHealth) or unstructured social support reported less consistent findings for physiological or mental health outcomes. However, differences in study designs, intervention characteristics and outcome measures preclude conclusions regarding the comparative effectiveness of these approaches. The evidence also suggests that longer programmes with progressive or transitional designs, providing intensive initial support that gradually decreases over time, may better promote empowerment and sustained self-management at home. The need to overcome emotional barriers that can hinder adherence to exercise, such as kinesiophobia and depression, highlights the importance of nurses moving beyond an exclusively physical approach by conducting comprehensive assessments and developing personalised care plans. The scarcity of follow-up periods exceeding 12 months also underscores the need to evaluate the impact of these interventions on long-term clinical outcomes, including mortality and hospital readmission rates. This review further highlights the marked geographical concentration of evidence in Asian populations and the frequent exclusion of older patients with frailty or limited digital access. Consequently, the findings apply mainly to the populations studied and cannot be generalised to all older adults with coronary heart disease. Despite the methodological heterogeneity of the included studies and the limitations inherent in the use of locally specific technological platforms, the findings support the need to develop and evaluate flexible, equitable and multicomponent nursing interventions to optimise comprehensive rehabilitation for older adults with coronary heart disease.

## Figures and Tables

**Table 1:** Main results of the included articles.

Author (Year) Country	Domain	Design	Population	Objective	Nursing Role	Intervention	Duration and Follow-up	Variables and Instruments	Main Outcomes
(Cao et al., 2023) [41] China	Psychological and emotional domain	RCT	*n* = 128 Mean age: 61.02 years; >70% hospitalised with CHD and type D personality	To examine the effects of an educational intervention on psychological health, health behaviours and quality of life	Direct delivery: nursing team	IG—Hospital phase: 2 face-to-face health education sessions to address misconceptions, set goals and develop coping strategies (diet, physical exercise and stress). Participants were provided with a manual and diary. IG—Home-based phase: 6 telephone follow-up calls to assess progress and address barriers. CG: Usual medical and nursing care.	Intervention: 12 weeks. Follow-up: 1 and 3 months.	Anxiety and depression (HADS) Quality of life (SAQ) Health-promoting behaviours (HPLP) Self-efficacy (GSES)	Anxiety-HADS: reduction in IG vs. CG (β = −1.84, 95% CI: −2.91 to −0.77, *p* < 0.05). Depression-HADS: reduction in IG vs. CG (β = −1.41, 95% CI: −2.27 to −0.55, *p* < 0.05). SAQ: Angina stability: β = +20.98 at 1 month and β = +29.23 at 3 months (*p* < 0.05). Physical activity limitation: β = +5.86 at 3 months (*p* < 0.05). Treatment satisfaction: β = +6.95 at 3 months (*p* < 0.05). HPLP total: improvement in IG at 1 month (β = +5.70) and 3 months (β = +11.50) (*p* < 0.05). GSES: improvement in IG at 3 months (β = +2.08, *p* < 0.05).
(Duan & Ren, 2023) [42] China	Biological and functional domain	RCT	*n* = 120 Mean age: 71.1 years, with CHD	To evaluate the effects of the NNN-linked care model on cardiac function, self-care capacity and quality of life	Direct delivery: nursing team	IG: NNN-linked model. Assessments based on NANDA-I diagnoses + setting NOC outcomes + implementation of NIC interventions. The care plan was monitored and adjusted according to patient progress. CG: Standard nursing care.	Intervention: 12 weeks. Follow-up: 3 months.	Cardiac function (LVEF, VO_2_ max, 6MWT) Self-care (ESCA) Quality of life (CQQC)	LVEF: IG 38.3% vs. CG 35.7% (*p* < 0.001). VO_2_ max: IG 17.9 ± 1.6 vs. CG 17.0 ± 1.4 (*p* < 0.01). 6MWT: IG 162.8 m ± 10.8 vs. CG 145.3 m ± 8.1 (*p* < 0.001). ESCA: IG 144.5 vs. CG 137.1 (*p* < 0.001). CQQC: IG 31.4 vs. CG 29.8 (*p* < 0.001).
(Guo et al., 2025) [43] China	Psychological and emotional domain	RCT	*n* = 86 Mean age: ~70 years, with CHD following PCI	To explore whether cardiac rehabilitation with psychological support reduces kinesiophobia	Direct delivery: mental health nurses + cardiac rehabilitation nurses	IG and CG—Physical phase: PE, ES, dietary management and medication management. IG—Psychological phase: Empathetic counselling, relaxation/breathing exercises/music therapy and self-motivation. Individualised care plans. CG: Standard cardiac rehabilitation protocol.	Intervention: 4–6 weeks. Follow-up: 2 months.	Anxiety (SAS) Depression (SDS) Exercise self-efficacy (MSES) Kinesiophobia (TSK-Heart) Quality of life (SF-36)	SAS: IG 25.71 vs. CG 27.95 (*p* = 0.0129). SDS: IG 30.98 vs. CG 34.30 (*p* = 0.0004). MSES total: IG 52.90 vs. CG 44.86 (*p* = 0.0092). TSK-Heart total: IG 33.88 vs. CG 41.32 (*p* < 0.001). SF-36 total: IG 85.43 vs. CG 79.21 (*p* = 0.0005).
(Hao et al., 2021) [44] China	Biological and functional domain	Quasi-experimental	*n* = 96 Mean age: >60 years, with CHD	To explore the effects of a community-based meticulous nursing care model on functional capacity, self-care and emotional well-being	Direct delivery/management: specialist nurses lead within the cardiac disease management team	IG: Meticulous home-based nursing care. Home visits (fortnightly initially, then monthly) + ES via WeChat + psychological intervention involving family members + guided, progressive PE. CG: Clinical monitoring and standard community care.	Intervention: 3 months. Follow-up: 3 months.	Functional capacity (6MWT) Self-care (ESCA) Quality of life (CQQC) Mood (POMS) Hope/expectations (HHI) Adherence to health-related behaviours (medication, diet, PE, follow-up visits)	6MWT: IG 326.82 m vs. CG 273.24 m (*p* < 0.001). ESCA: improvements in IG (*p* < 0.001) across all dimensions (self-care ability, self-concept, responsibility and level of knowledge). CQQC: IG 127.84 vs. CG 103.69 (*p* < 0.001). POMS: reduction in negative emotions, IG 13.28 vs. CG 19.87 (*p* < 0.001). HHI: IG 37.99 vs. CG 32.83 (*p* < 0.001). Adherence to health-related behaviours (*p* < 0.05): higher in IG for medication (IG 89.5% vs. CG 60.4%), diet (IG 85.4% vs. CG 66.6%), follow-up visits (IG 79.1% vs. CG 60.4%) and PE (IG 72.9% vs. CG 52%).
(Heidari et al., 2024) [45] Irán	Social, educational and self-care domain	Quasi-experimental	*n* = 70 Mean age: IG 59.24 ± 5.22 years; CG 64.44 ± 3.26 years, following AMI (ICU)	To explore the impact of Orem’s self-care model on quality of life	Direct delivery: nursing team	IG: Programme based on Orem’s self-care model. Four individualised sessions covering the condition, diet, medication adherence, PA and self-care. Participants were provided with an educational leaflet. CG: Usual medical and nursing care in the ICU.	Intervention: 8 weeks. Follow-up: 2 weeks and 2 months.	Quality of life (MacNew) Self-care (Myocardial Infarction Self-Care Brief)	MacNew: IG 58.94 to 168.43 vs. CG 54.69 to 72.00 (*p* < 0.001). Myocardial Infarction Self-Care Ability Questionnaire: Self-care skills: IG 102.10 vs. CG 53.03 (*p* < 0.005). Self-care readiness: IG 31.60 vs. CG 17.46 (*p* < 0.005). Self-care knowledge: IG 112.77 vs. CG 41.74 (*p* < 0.005).
(Hua et al., 2024) [46] China	Psychological and emotional domain	RCT	*n* = 85 Mean age: ~60.5 years, following coronary stent implantation	To evaluate the effectiveness of telephone follow-up based on empowerment theory in improving resilience and quality of life	Direct delivery: nursing team	IG—At discharge: 1 session to assess potential and set rehabilitation goals. IG—Home-based phase: Weekly telephone follow-up based on five steps (identifying problems, expressing emotions, setting goals, planning and evaluation). CG: Routine care and ES.	Intervention: 4 weeks. Follow-up: 1 month.	Resilience (CD-RISC) Quality of life (SF-36)	CD-RISC: IG 65.79 ± 13.40 vs. CG 58.74 ± 12.77 (*p* < 0.05). SF-36: improvement in IG, 67.06 ± 14.83 vs. CG 60.76 ± 11.13 (*p* = 0.029). No SDiff in physical functioning, bodily pain, vitality or mental health.
(Li et al., 2022) [47] China	Social, educational and self-care domain	RCT	*n* = 89 Mean age: 60.2 ± 11.3 years, hospitalised with CHD	To explore the effectiveness of micro-lecture-based education in improving self-management capacity and promoting lifestyle changes	Direct delivery: nursing team	IG—Hospital phase: Viewing of short educational videos (micro-lectures, twice daily) on the condition and lifestyle. IG—Home-based phase: Self-regulated home-based PE using the Borg scale and a recording diary. CG: Traditional ES using leaflets.	Intervention: 12 weeks. Follow-up: 4 and 12 weeks.	Self-management (CSMS) Smoking (smoking status) PE: frequency, duration and type (diary) Functional capacity (6MWT)	CSMS: IG 3.84 vs. CG 2.78 (*p* < 0.001). Smoking relapse: IG 6% vs. CG 43% (*p* = 0.028). Exercise frequency (≥3 times/week): IG 85% vs. CG 31% (*p* < 0.001). 6MWT: IG 495.55 vs. CG 370.24 (*p* < 0.001).
(Pedersen et al., 2025) [48] Dinamarca	Social, educational and self-care domain	Mixed- Methods	*n* = 117 Mean age: ≥65 years, with ischaemic heart disease	To clarify the effective mechanisms and barriers associated with a peer mentor support intervention	Coordination and supervision: nurses train and supervise peer mentors who deliver the intervention	IG: Support from volunteer peer mentors. Flexible face-to-face and telephone contact (mean of 5 contacts). Mentors shared their own experiences to encourage attendance at rehabilitation, promote lifestyle improvements and provide emotional support.	Intervention: 24 weeks. Follow-up: 12 and 24 weeks.	Cardiac rehabilitation attendance Self-efficacy (GSE) Anxiety and depression (HADS) Quality of life (HeartQoL) Diet (HeartDiet) PA (>6 times/week) Satisfaction and perceived effect (qualitative)	AF: IG 20% to 27% vs. CG 19% to 22%; no SDiff. Healthy diet: IG 1.9% to 7.4% vs. CG 3.6% to 3.8%; no significant differences. HADS: no SDiff in anxiety or depression. GSE and HeartQoL: no SDiff. Satisfaction (IG): 92%. Perceived effect (IG): 74%.
(Roshandel et al., 2021) [49] Irán	Social, educational and self-care domain	RCT	*n* = 30 Age: 60–85 years, with CHD	To investigate the effects of peer patient education on self-care, treatment adherence and quality of life	Coordination and supervision: nursing team trains and supervises peer patients	IG: ES delivered by peer patients via WhatsApp + educational videos lasting 20 min each. CG: Routine ES + standard videos.	Intervention: 1 month. Follow-up: 1 month.	Self-care (34-item self-care questionnaire) Treatment adherence (adherence questionnaire for patients with chronic conditions) Quality of life (SF-12)	Self-care questionnaire: increase in IG, 18.46 ± 7.93 vs. CG 7.06 ± 3.71 (*p* < 0.001). Adherence questionnaire: increase in IG, 10.93 ± 4.75 vs. CG 3.83 ± 1.55 (*p* < 0.001). SF-12: increase in IG, 8.20 ± 4.69 vs. CG 2.73 ± 1.62 (*p* < 0.001).
(Snaid & Qibalat, 2022) [50] Jordania	Biological and functional domain	RCT	*n* = 200 Mean age: 58.1 ± 8.8 and 59.1 ± 8.3 years, with CHD	To examine the effects of a comprehensive nursing intervention (CBT + psychosocial counselling) on cardiopulmonary function and emotional well-being	Direct delivery: nursing team	IG: Standard care + weekly ES + TCC (twice weekly) + telephone follow-up for 6 months after discharge. CG: Standard nursing care.	Intervention: 6 months. Follow-up: 6 months.	Anxiety (SAS) Depression (SDS) Quality of life (SF-36) Cardiopulmonary function (Tei index and 6MWT)	SAS: IG 49.24 ± 25.22 vs. CG 67.56 ± 28.64 (*p* < 0.05). SDS: IG 49.24 ± 28.54 vs. CG 60.37 ± 30.22 (*p* < 0.05). SF-36: higher scores in IG (*p* < 0.001). 6MWT: IG 427.88 m vs. CG 422.61 m (*p* = 0.005). Tei index: IG 0.42 ± 0.30 vs. CG 0.54 ± 0.32 (*p* = 0.041).
(Su & Yu, 2021) [51] China	Social, educational and self-care domain	RCT	*n* = 146 patients Mean age: 55.78 ± 7.14 years, hospitalised with CHD	To evaluate the effects of a nurse-led eHealth-based cardiac rehabilitation programme (NeCR) on health behaviours, self-efficacy, anxiety, depression, quality of life, physiological risk parameters and unplanned healthcare utilisation	Direct delivery: registered nurse with cardiovascular nursing experience; peer patients provide support	IG: Web-based platform (for health education, daily recording of achievements and pedometer use) + participation in a nurse-moderated WeChat group, with peer support and motivational interviewing. CG: Traditional face-to-face health education session + pedometer use.	Intervention: 12 weeks. Follow-up: 6 and 12 weeks.	PA (steps/day, sedentary time, MET) Lifestyle (HPLP-II) Cardiac self-efficacy (CSES) Health-related quality of life (MacNew) Psychological well-being (DASS-21) Clinical parameters (BP, BMI and WC)	Steps/day: IG 9502.29 vs. CG 7298.04 (*p* = 0.006). Sedentary time (min/week): IG 1239.77 vs. CG 2010.73 (*p* = 0.004). MET: IG 2796 vs. CG 1833 (*p* = 0.015). HPLP-II: IG 110.35 vs. CG 95.00 (*p* < 0.001). CSES: IG 3.31 vs. CG 2.79 (*p* = 0.005). MacNew: physical subscale β = 0.45 (*p* = 0.049) and social subscale β = 0.54 (*p* = 0.025); no SDiff in the overall score. DASS-21: no SDiff. DBP, BMI and WC: no SDiff.
(Zou et al., 2024) [52] China	Psychological and emotional domain	RCT	*n* = 178 Mean age: 58.7 ± 8.9 years, with ACS	To examine the effects of a mindfulness-based psychological and behavioural intervention (MCARE) on psychological distress, stress, quality of life and cardiovascular risk factors	Direct delivery: nursing team	IG—Hospital phase: 1 face-to-face ES session on the condition and mindfulness practice. IG—Home-based phase: 5 sessions/week via WeChat (psychoeducation and lifestyle) + home-based mindfulness practice (10–20 min/day, 6 days/week). Two telephone calls to review progress and provide support. MCARE programme. CG: Routine care.	Intervention: 6 weeks. Follow-up: 6 weeks (T1) and 12 weeks (T2).	Depression (PHQ-9) Anxiety (GAD-7) Psychological stress (PSS-10) Health-related quality of life (HeartQoL) Cardiovascular risk factors (IPAQ-S, HPLP-II, smoking, BMI, PA, lipids and blood glucose)	PHQ-9: reduction in IG at T1 (β = −2.01) and T2 (β = −2.08) (*p* < 0.001). GAD-7: reduction in IG at T1 (β = −1.02, *p* < 0.001) and T2 (β = −0.93, *p* = 0.002). PSS-10: reduction in IG at T1 (β = −1.18, *p* < 0.001) and T2 (β = −1.26, *p* = 0.001). HeartQoL: Physical: T1 (β = 0.088, *p* = 0.03). Emotional: T1 (β = 0.294, *p* < 0.001); T2 (β = 0.249, *p* = 0.001). Overall: T1 (β = 0.147, *p* < 0.001); T2 (no SDiff). Risk factors: SBP: reduction in IG at T2 (β = −3.32, *p* = 0.01). Dietary behaviour: T2 (β = 0.069, *p* = 0.04). PA level: T2 (β = 177.54, *p* = 0.01). Smoking, BMI, PAD, lipids and blood glucose: no SDiff.

6MWT, Six-Minute Walk Test; PA, physical activity; ADL, activities of daily living; WC, waist circumference; CD-RISC, Connor-Davidson Resilience Scale; CG, control group; CHD, coronary heart disease; CQQC, Chinese Questionnaire of Quality of Life in Patients with Cardiovascular Disease; CSES, Cardiac Self-Efficacy Scale; CSMS, Coronary Self-Management Scale; TC, total cholesterol; DASS-21, Depression Anxiety Stress Scales-21; SD, standard deviation; SDiff, significant differences; RCT, randomised controlled trial; PE, physical exercise; HE, health education; ESCA, Exercise of Self-Care Agency Scale; HR, heart rate; GAD-7, Generalized Anxiety Disorder-7; GSE/GSES, General Self-Efficacy Scale; HADS, Hospital Anxiety and Depression Scale; HeartQoL, Heart-related Quality of Life Questionnaire; HHI, Herth Hope Index; HPLP-II, Health-Promoting Lifestyle Profile II; AMI, acute myocardial infarction; IG, intervention group; IHD, ischaemic heart disease; BMI, body mass index; IPAQ, International Physical Activity Questionnaire; IPAQ-SF, International Physical Activity Questionnaire-Short Form; LVEF, left ventricular ejection fraction; MacNew, MacNew Heart Disease Health-Related Quality of Life Questionnaire; MET, metabolic equivalents; MSES, Multidimensional Sport Self-Efficacy Scale; DBP, diastolic blood pressure; SBP, systolic blood pressure; PCI, percutaneous coronary intervention; PHQ-9, Patient Health Questionnaire-9; POMS, Profile of Mood States; PSS-10, Perceived Stress Scale-10; CR, cardiac rehabilitation; SAQ, Seattle Angina Questionnaire; SAS, Self-Rating Anxiety Scale; ACS, acute coronary syndrome; SDS, Self-Rating Depression Scale; SF-12, Short Form Health Survey-12; SF-36, Short Form Health Survey-36; T1, immediately post-intervention; T2, follow-up; BP, blood pressure; CBT, cognitive behavioural therapy; TSK-Heart, Tampa Scale for Kinesiophobia-Heart; ICU, intensive care unit; VO_2_ max, maximal oxygen uptake. Outcomes values are reported as in the original studies and are not comparable across different instruments.

**Table 2:** Synthesis of intervention characteristics and outcome domains across the included studies (*n* = 12).

Dimension	Category	*n*	Studies
**Clinical population**	Coronary heart disease (general)	7	[41,42,44,47,48,49,51]
	After percutaneous coronary intervention	2	[43,46]
	Acute coronary syndrome	1	[52]
	Acute myocardial infarction (ICU)	1	[45]
	Stable angina	1	[50]
**Mean age**	≥60 years	9	[41,42,43,44,45,46,47,48,49]
	<60 years	3	[50,51,52]
**Duration**	≤8 weeks	5	[43,45,46,49,52]
	12 weeks/3 months	5	[41,42,44,47,51]
	6 months	2	[48,50]
**Care setting**	Hospital to home	6	[41,42,47,50,51,52]
	Home after discharge	3	[46,48,49]
	Hospital (continuation not reported)	2	[43,45]
	Community	1	[44]
**Mode of delivery**	Blended (face-to-face and remote)	9	[41,42,43,44,45,47,48,50,52]
	Digital only	2	[49,51]
	Telephone only	1	[46]
**Nursing role**	Direct delivery	10	[41,42,43,44,45,46,47,50,51,52]
	Coordination and supervision of peers	2	[48,49]
**Outcome domains (instruments)**	Functional capacity (6MWT, VO_2_ max)	4	[42,44,47,50]
	Cardiovascular and physiological parameters (LVEF, Tei index, BP, BMI, WC, lipids, glucose)	4	[42,50,51,52]
	Anxiety and depression (HADS, SAS, SDS, DASS-21, PHQ-9, GAD-7)	6	[41,43,48,50,51,52]
	Kinesiophobia, stress, resilience and mood (TSK-Heart, PSS-10, CD-RISC, POMS, HHI)	4	[43,44,46,52]
	Self-care and health behaviours (ESCA, CSMS, HPLP-II, IPAQ-SF, specific questionnaires)	8	[42,44,45,47,48,49,51,52]
	Self-efficacy (GSES, MSES, GSE, CSES)	4	[41,43,48,51]
	Health-related quality of life, generic (SF-36, SF-12)	4	[43,46,49,50]
	Health-related quality of life, disease-specific (SAQ, CQQC, MacNew, HeartQoL)	7	[41,42,44,45,48,51,52]
	Healthcare use and patient experience	5	[41,44,48,51,52]

Categories are mutually exclusive except for outcome domains, as most studies assessed more than one domain. Instruments are listed as reported in the original studies; values obtained with different instruments are not directly comparable. Abbreviations as in Table 1.

## Data Availability

No new data were created or analyzed in this study. Data sharing is not applicable to this article.

## Appendix A. Search Terms and Search Strategies

### Appendix A.1

**Table A1:** Structured Language Standardisation Table.

Keywords (English)	Keywords (Spanish)	Medical Subject Headings (MeSH)
Chronic ischemic heart disease	Enfermedad coronaria	*Coronary Disease, Myocardial ischemia*
Coronary heart disease		
Acute coronary syndrome		
Myocardial ischemia		
Elderly	Adultos mayores	*Aged*
Aged		
Senior adults		
Older adults		
Older people		
Older person *		
Aging		
Intervention	Intervención	*Therapeutics*
Program *		
Treatment		
Therapy		
Strategy		
Method		
Care		
Nurse *	Enfermería	*Nurses*
Nurse interventions		
Nurse programs		
Functional rehabilitation	Rehabilitación	*Rehabilitation*
Physical rehabilitation		
Cardiac rehabilitation		
Comprehensive rehabilitation		
Motor rehabilitation		
Functional recovery		
Functional capacity		
Functional mobility		
Activities of daily living (ADL)		
Quality of life		
Community-based rehabilitation		
Post-hospital rehabilitation		

The asterisk (*) indicates a truncation operator used to retrieve different word forms sharing the same root.

### Appendix A.2

To exclude articles focusing on pharmacological approaches from the database searches, the Boolean operator NOT was used in combination with the following terms.

**Table A2:** Search Terms Excluded Using the Boolean Operator NOT.

Keywords (English)	Keywords (Spanish)	Medical Subject Headings (MeSH)
Pharmacological treatment	Tratamiento farmacológico	*Drug therapy*
Medication		
Pharmacotherapy		

### Appendix A.3

**Table A3:** Search strategy adapted to each database.

Data Base	Search Strategy (Title-Abs-Key)	Results
PubMed	((((((((((Chronic ischemic heart disease[Title/Abstract]) OR (Coronary heart disease[Title/Abstract])) OR (Acute coronary syndrome[Title/Abstract])) OR (Post-heart attack[Title/Abstract])) OR (Myocardial ischemia[Title/Abstract])) OR (coronary disease[MeSH Terms])) OR (Myocardial ischemia[MeSH Terms])) AND (((((((Elderly[Title/Abstract]) OR (Aged[Title/Abstract])) OR (Senior adults[Title/Abstract])) OR (Older adults[Title/Abstract])) OR (Older people[Title/Abstract])) OR (Aging[Title/Abstract])) OR (aged[MeSH Terms])))) AND ((((((((((Intervention[Title/Abstract]) OR (Program[Title/Abstract])) OR (Treatment[Title/Abstract])) OR (Therapy[Title/Abstract])) OR (Strategy[Title/Abstract])) OR (Method[Title/Abstract])) OR (Care[Title/Abstract]))) OR (therapeutics[MeSH Terms]))) AND ((((nurses[MeSH Terms]) OR (nurse*[Title/Abstract])) OR (“nurse interventions”[Title/Abstract])) OR (“nurse programs”[Title/Abstract])) AND (((((((((((((Functional rehabilitation[Title/Abstract]) OR (Physical rehabilitation[Title/Abstract])) OR (Cardiac rehabilitation[Title/Abstract])) OR (Comprehensive rehabilitation[Title/Abstract])) OR (Motor rehabilitation[Title/Abstract])) OR (Functional recovery[Title/Abstract])) OR (Functional capacity[Title/Abstract])) OR (Functional mobility[Title/Abstract])) OR (Activities of daily living[Title/Abstract])) OR (Quality of life[Title/Abstract])) OR (Community-based rehabilitation[Title/Abstract])) OR (Post-hospital rehabilitation[Title/Abstract])) OR (rehabilitation[MeSH Terms]))) NOT ((“Drug Therapy”[MeSH Terms] OR “Pharmacological treatment”[Title/Abstract] OR “Medication”[Title/Abstract] OR “Pharmacotherapy”[Title/Abstract]))	157
CINHAL	(TI “Chronic ischemic heart disease” OR AB “Chronic ischemic heart disease” OR TI “Coronary heart disease” OR AB “Coronary heart disease” OR TI “Acute coronary syndrome” OR AB “Acute coronary syndrome” OR TI “Post-heart attack” OR AB “Post-heart attack” OR TI “Myocardial ischemia” OR AB “Myocardial ischemia” OR (MH “Coronary Disease+”) OR (MH “Myocardial Ischemia+”)) AND (TI Elderly OR AB Elderly OR TI Aged OR AB Aged OR TI “Senior adults” OR AB “Senior adults” OR TI “Older adults” OR AB “Older adults” OR TI “Older people” OR AB “Older people” OR TI Aging OR AB Aging OR (MH “Aged+”)) AND (TI Intervention OR AB Intervention OR TI Program* OR AB Program* OR TI Treatment OR AB Treatment OR TI Therapy OR AB Therapy OR TI Strategy OR AB Strategy OR TI Method OR AB Method OR TI Care OR AB Care OR (MH “Therapeutic Outcomes+”)) AND (TI nurse* OR AB nurse* OR TI “nurse interventions” OR AB “nurse interventions” OR TI “nurse programs” OR AB “nurse programs” OR (MH “Nurses+”) OR (MH “Nursing Interventions+”)) AND (TI “Functional rehabilitation” OR AB “Functional rehabilitation” OR TI “Physical rehabilitation” OR AB “Physical rehabilitation” OR TI “Cardiac rehabilitation” OR AB “Cardiac rehabilitation” OR TI “Comprehensive rehabilitation” OR AB “Comprehensive rehabilitation” OR TI “Motor rehabilitation” OR AB “Motor rehabilitation” OR TI “Functional recovery” OR AB “Functional recovery” OR TI “Functional capacity” OR AB “Functional capacity” OR TI “Functional mobility” OR AB “Functional mobility” OR TI “Activities of daily living” OR AB “Activities of daily living” OR TI “Quality of life” OR AB “Quality of life” OR (MH “Rehabilitation+”) OR (MH “Activities of Daily Living+”) OR (MH “Quality of Life+”)) NOT (TI “Pharmacological treatment” OR AB “Pharmacological treatment” OR TI Medication OR AB Medication OR TI Pharmacotherapy OR AB Pharmacotherapy OR (MH “Drug Therapy+”))	303
SCOPUS	(((((((((TITLE-ABS (“Chronic ischemic heart disease”) OR TITLE-ABS (“Coronary heart disease”) OR TITLE-ABS (“Acute coronary syndrome”) OR TITLE-ABS (“Post-heart attack”) OR TITLE-ABS (“Myocardial ischemia”) OR KEY (“coronary disease”) OR KEY (“Myocardial ischemia”)) AND (TITLE-ABS (Elderly) OR TITLE-ABS (Aged) OR TITLE-ABS (“Senior adults”) OR TITLE-ABS (“Older adults”) OR TITLE-ABS (“Older people”) OR TITLE-ABS (Aging) OR KEY (aged)))) AND (TITLE-ABS (Intervention) OR TITLE-ABS (Program) OR TITLE-ABS (Treatment) OR TITLE-ABS (Therapy) OR TITLE-ABS (Strategy) OR TITLE-ABS (Method) OR TITLE-ABS (Care) OR KEY (therapeutics)))) AND (KEY (nurses) OR TITLE-ABS (nurse*) OR TITLE-ABS (“nurse interventions”) OR TITLE-ABS (“nurse programs”)))) AND (TITLE-ABS (“Functional rehabilitation”) OR TITLE-ABS (“Physical rehabilitation”) OR TITLE-ABS (“Cardiac rehabilitation”) OR TITLE-ABS (“Comprehensive rehabilitation”) OR TITLE-ABS (“Motor rehabilitation”) OR TITLE-ABS (“Functional recovery”) OR TITLE-ABS (“Functional capacity”) OR TITLE-ABS (“Functional mobility”) OR TITLE-ABS (“Activities of daily living”) OR TITLE-ABS (“Quality of life”) OR TITLE-ABS (“Community-based rehabilitation”) OR TITLE-ABS (“Post-hospital rehabilitation”) OR KEY (rehabilitation)))) AND NOT (KEY (“Drug Therapy”) OR TITLE-ABS (“Pharmacological treatment”) OR TITLE-ABS (Medication) OR TITLE-ABS (Pharmacotherapy))	111
Web of Science	TS=(“Chronic ischemic heart disease” OR “Coronary heart disease” OR “Acute coronary syndrome” OR “Post-heart attack” OR “Myocardial ischemia” OR “coronary disease”) AND TS=(Elderly OR Aged OR “Senior adults” OR “Older adults” OR “Older people” OR Aging) AND TS=(Intervention OR Program OR Treatment OR Therapy OR Strategy OR Method OR Care OR therapeutics) AND TS=(nurses OR nurse* OR “nurse interventions” OR “nurse programs”) AND TS=(“Functional rehabilitation” OR “Physical rehabilitation” OR “Cardiac rehabilitation” OR “Comprehensive rehabilitation” OR “Motor rehabilitation” OR “Functional recovery” OR “Functional capacity” OR “Functional mobility” OR “Activities of daily living” OR “Quality of life” OR “Community-based rehabilitation” OR “Post-hospital rehabilitation” OR rehabilitation) NOT TS=(“Drug Therapy” OR “Pharmacological treatment” OR Medication OR Pharmacotherapy)	569
Cochrane	((((((((((((“Chronic ischemic heart disease”:ti,ab,kw) OR (“Coronary heart disease”:ti,ab,kw)) OR (“Acute coronary syndrome”:ti,ab,kw)) OR (“Post-heart attack”:ti,ab,kw)) OR (“Myocardial ischemia”:ti,ab,kw)) OR [mh “coronary disease”]) OR [mh “Myocardial ischemia”])) AND ((((((((Elderly:ti,ab,kw) OR (Aged:ti,ab,kw)) OR (“Senior adults”:ti,ab,kw)) OR (“Older adults”:ti,ab,kw)) OR (“Older people”:ti,ab,kw)) OR (Aging:ti,ab,kw)) OR [mh aged]))) AND (((((((((((Intervention:ti,ab,kw) OR (Program*:ti,ab,kw)) OR (Treatment:ti,ab,kw)) OR (Therapy:ti,ab,kw)) OR (Strategy:ti,ab,kw)) OR (Method:ti,ab,kw)) OR (Care:ti,ab,kw)))) OR [mh therapeutics]))) AND (((((nurses:ti,ab,kw) OR (nurse*:ti,ab,kw)) OR (“nurse interventions”:ti,ab,kw)) OR (“nurse programs”:ti,ab,kw)) OR [mh nurses]))) AND ((((((((((((((“Functional rehabilitation”:ti,ab,kw) OR (“Physical rehabilitation”:ti,ab,kw)) OR (“Cardiac rehabilitation”:ti,ab,kw)) OR (“Comprehensive rehabilitation”:ti,ab,kw)) OR (“Motor rehabilitation”:ti,ab,kw)) OR (“Functional recovery”:ti,ab,kw)) OR (“Functional capacity”:ti,ab,kw)) OR (“Functional mobility”:ti,ab,kw)) OR (“Activities of daily living”:ti,ab,kw)) OR (“Quality of life”:ti,ab,kw)) OR (“Community-based rehabilitation”:ti,ab,kw)) OR (“Post-hospital rehabilitation”:ti,ab,kw)) OR [mh rehabilitation])) NOT (([mh “Drug Therapy”] OR (“Pharmacological treatment”:ti,ab,kw) OR (Medication:ti,ab,kw) OR (Pharmacotherapy:ti,ab,kw)))	123
TOTAL		1263

The asterisk (*) is used as a truncation wildcard in the search strategy.

## Appendix B. Quality Assessment of Included Studies

### Appendix B.1

**Table A4:** Quality Assessment Table Using the JBI Critical Appraisal Tool for Randomised Controlled Trials (RCTs).

	Was True Randomization Used for Assignment of Participants to Treatment Groups?	Was Allocation to Treatment Groups Concealed?	Were Treatment Groups Similar at the Baseline?	Were Participants Blind to Treatment Assignment?	Were Those Delivering the Treatment Blind to Treatment Assignment?	Were Treatment Groups Treated Identically Other than the Intervention of Interest?	Were Outcome Assessors Blind to Treatment Assignment?	Were Outcomes Measured in the Same Way for Treatment Groups?	Were Outcomes Measured in a Reliable Way?	Was Follow up Complete and If Not, Were Differences Between Groups in Terms of Their Follow up Adequately Described and Analysed?	Were Participants Analysed in the Groups to Which They Were Randomized?	Was Appropriate Statistical Analysis Used?	Was the Trial Design Appropriate and Any Deviations from the Standard RCT Design (Individual Randomization, Parallel Groups) Accounted for in the Conduct and Analysis of the Trial?	Score
(Cao et al., 2023) [41]	Yes	Yes	Yes	No	No	-	Yes	Yes	Yes	Yes	Yes	Yes	Yes	10/13
(Duan & Ren, 2023) [42]	Yes	-	Yes	No	No	-	Yes	Yes	Yes	Yes	Yes	Yes	Yes	9/13
(Guo et al., 2025) [43]	Yes	No	Yes	No	No	-	Yes	Yes	Yes	Yes	Yes	Yes	Yes	9/13
(Hua et al., 2024) [46]	Yes	-	Yes	Yes	No	Yes	Yes	Yes	Yes	Yes	Yes	Yes	Yes	11/13
(Li et al., 2022) [47]	Yes	Yes	Yes	No	No	Yes	Yes	Yes	Yes	Yes	Yes	Yes	Yes	11/13
(Roshandel et al., 2021) [49]	Yes	-	Yes	No	No	-	Yes	Yes	Yes	Yes	Yes	Yes	Yes	9/13
(Snaid & Qibalat, 2022) [50]	Yes	-	Yes	No	No	-	Yes	Yes	Yes	Yes	Yes	Yes	Yes	9/13
(Su & Yu, 2021) [51]	Yes	Yes	Yes	No	No	Yes	Yes	Yes	Yes	Yes	Yes	Yes	Yes	11/13
(Zou et al., 2024) [52]	Yes	Yes	Yes	No	No	Yes	Yes	Yes	Yes	Yes	Yes	Yes	Yes	11/13

### Appendix B.2

**Table A5:** Methodological Quality Assessment Table Using the JBI Critical Appraisal Tool for Quasi-Experimental Studies.

	Is It Clear in the Study What Is the “Cause” and What Is the “Effect”?	Was There a Control Group?	Were Participants Included in Any Comparisons Similar?	Were the Participants Included in Any Comparisons Receiving Similar Treatment/Care, Other than the Exposure or Intervention of Interest?	Were There Multiple Measurements of the Outcome, Both Pre and Post the Intervention/Exposure?	Were the Outcomes of Participants Included in Any Comparisons Measured in the Same Way?	Were Outcomes Measured in a Reliable Way?	Was Follow-up Complete and If Not, Were Differences Between Groups in Terms of Their Follow-up Adequately Described and Analyzed?	Was Appropriate Statistical Analysis Used?	Score
(Hao et al., 2021) [44]	Yes	Yes	Yes	Yes	No	Yes	Yes	Yes	Yes	8/9
(Heidari et al., 2024) [45]	Yes	Yes	Yes	Yes	Yes	Yes	Yes	-	Yes	8/9

### Appendix B.3

**Table A6:** Methodological Quality Assessment Table Using the Mixed Methods Appraisal Tool (MMAT) for Mixed-Method Studies.

	Is There an Adequate Rationale for Using a Mixed Methods Design to Address the Research Question?	Are the Different Components of the Study Effectively Integrated to Answer the Research Question?	Are the Outputs of the Integration of Qualitative and Quantitative Components Adequately Interpreted?	Are Divergences and Inconsistencies Between Quantitative and Qualitative Results Adequately Addressed?	Do the Different Components of the Study Adhere to the Quality Criteria of Each Tradition of the Methods Involved?	Score
(Pedersen et al., 2025) [48]	Yes	Yes	Yes	Yes	Yes	5/5
